# Pulmonary embolism due to an intracardiac thrombosis in a patient affected by Behçet’s disease: a case report

**DOI:** 10.1093/ehjcr/ytae467

**Published:** 2024-09-03

**Authors:** Valeria Ambrosino, Francesca De Marco, Gabriele Valli, Maria Pia Ruggieri, Sergio Morelli

**Affiliations:** Postgraduate School of Internal Medicine, Sapienza University of Rome, 00185 Rome, Italy; Emergency Department, San Giovanni Addolorata Hospital, 00184 Rome, Italy; Emergency Department, San Giovanni Addolorata Hospital, 00184 Rome, Italy; Emergency Department, San Giovanni Addolorata Hospital, 00184 Rome, Italy; Postgraduate School of Internal Medicine, Sapienza University of Rome, 00185 Rome, Italy

**Keywords:** Behçet’s disease, Pulmonary embolism, Intracardiac thrombosis, Trans-thoracic echocardiogram, Case report

## Abstract

**Background:**

Behçet’s disease is an inflammatory condition, caused by vasculitis of big and small veins and arteries in which, although vascular inflammation is the basis of disease, cardiac involvement is rare. We present a rare case of a man, affected by Behçet’s disease, with pulmonary embolism due to a floating thrombus in the right ventricle.

**Case summary:**

We report a case of a 36-year-old man admitted to emergency department due to dyspnoea and haemoptysis. He had already been diagnosed with Behçet’s disease, and he was in therapy with low doses of azathioprine and prednisone from three months. Thorax CT scan detected pulmonary embolism with pulmonary infraction. No evidence of deep vein thrombosis was found. The echocardiogram pointed out a floating mass of at least 30 mm in the right ventricle. Cardiac magnetic resonance confirmed the diagnosis of right ventricle thrombosis. On the hypothesis of an inflammatory genesis of the thrombosis, immunosuppressive drugs and anticoagulation with vitamin K antagonist were prescribed. The patient underwent echocardiograms every 3 weeks, and the mass disappeared 5 months later.

**Discussion:**

Behçet’s disease is a systemic inflammatory disorder that often affects vessels and rarely the heart. Thrombosis can be the only clinical feature of primary or relapsing events with also atypical origin site. Thrombosis suggests a high inflammatory status that needs to be balanced with the right immunosuppressive therapy, associated to anticoagulation.

**Learning points**

- Thrombosis, in Behçet’s disease, is due to endothelial dysfunction and inflammatory activity of the syndrome itself; for that, anticoagulation and immunosuppression should be used in a synergic manner to be effective.
- An accurate imaging and a multisystemic approach are necessary in presence of pulmonary thromboembolism due to atypical thrombosis: it can be the only sign of a systemic disorder, and finding the underlying cause can improve earlier and more effective therapies.

## Introduction

Behçet’s disease (BD) is a rare immune-mediated disorder that affects ∼80–370 out of every 100 000 people in Turkey and 13–20 out of every 100 000 people in other countries.^1,2^ It is characterized by mucocutaneous involvement, including recurrent oral and genital ulcerations, uveitis, and systemic vasculitis in both small and large vessels. There are no definitive laboratory tests to diagnose BD, so the diagnosis is based on clinical criteria.^1^ The histopathological hallmark of BD is vasculitis in large, medium, and small vessels, which leads to endothelial dysfunction and a pro-coagulant state. The exact cause of BD is still unknown, but genetics and environmental factors could play a role. People who carry the HLA-B51/B5 gene are considered to be at a higher risk for developing BD compared to those who do not have the gene.^2^ The prevalence of BD is highest in Southeast Asia and along the ‘Silk Road’, where there is a higher prevalence among males; in most other countries, there is an equal distribution between males and females.^2^ Cardiovascular involvement in BD is estimated to occur in 7%–46% of cases.^2^

Arterial involvement, which is more commonly characterized by aneurysms and rarely by arterial thrombosis, is less common than venous involvement, which accounts for 77%–95% of all the vascular features of BD. In a retrospective study by Wu X *et al*., 93 BD patients with cardiovascular involvement were evaluated, and the most common manifestation was peripheral vein thrombosis (86%). Other features included vena cava thrombosis (30.1%), pulmonary thromboembolism (15.1%), cerebral venous thrombosis (12.9%), intracardiac thrombosis (8.6%), Budd–Chiari syndrome (7.5%), and renal vein thrombosis (4.3%).^3^ Cardiac involvement alone is less prevalent (6%) and can present as pericarditis (38.5%), endocarditis (26.9%), and intracardiac thrombosis (19.2%), mainly in the right heart (78%).^4^ Currently, there are no specific guidelines for managing cardiac involvement in BD and there is limited evidence on the appropriate treatment for thrombotic complications of this disease.^5^

## Summary figure

**Figure:**
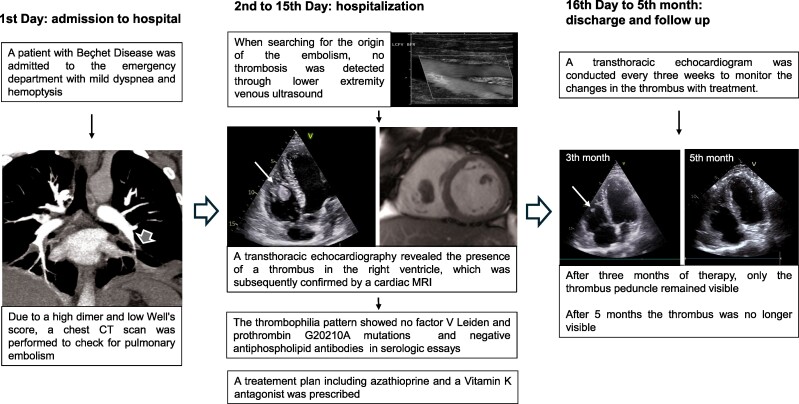


## Case presentation

We present a case study of a 36-year-old man who was admitted to the emergency department of a central teaching hospital in Rome, Italy, with a 3-day history of dyspnoea, haemoptysis, and pleural pain. He had previously been diagnosed with BD, with a history of acne-like folliculitis on his back, oral ulcerative lesions, and relapsing orchitis. There was no reported ocular involvement. The patient had been diagnosed with BD three months prior, based on clinical features and laboratory findings, and was being treated with prednisone (25 mg per day) and azathioprine (50 mg per day). Genetic testing for the HLA-B51 mutation was negative. Serological tests showed no evidence of auto-antibodies, with negative results for anti-nuclear antibodies (ANA), rheumatoid factor (RF), anti-citrulline antibodies (anti-CCP), and antiphospholipid antibodies (APS). Upon admission, the patient had mild dyspnoea without wheezes or crackles and reported acute left basal pain when taking deep breaths. He had normal body temperature, blood pressure, peripheral oxygen levels on room air, and a sinus rhythm with a heart rate of 75 b.p.m. on the ECG. Blood tests showed a normal arterial blood gas sample, leukocytosis with neutrophilia, high D-dimer levels, slightly elevated C-reactive protein levels, normal high-sensitivity troponin levels, and normal kidney and liver function (*Table 1*). Sputum culture and *Mycobacterium tuberculosis* testing were negative.

**Table 1:** Patient’s blood test, serological auto-antibodies screen, and arterial blood gas test at the BD diagnosis, at the admission in emergency department and 5 months later

	Normal values	BD diagnosis	At the entrance	5 months later
White cells count, cell/mm^3^ (%Neu)	4000–8000 cell/mm^3^	14.090 (83%)	13.000 (88%)	7.960 (64%)
CRP, mg/dL	<0.5 mg/dL	11.04	8	0.11
D-dimer, mg/L	<0.05 mg/L	0.05	1.8	0.02
Creatinine, mg/dL	<1.2 mg/dL	1.0	1.1	0.93
ALT, U/L	<48 U/L	9	34	19
AST, U/L	<55 U/L	12	27	11
RF, U/mL	<20 U/mL	12	16	–
Anti-CCP, U/mL	<7.0 U/mL	5.2	3.4	–
ANA, U	≤1:80 U	Negative	Negative	–
aCL IgG, U/mL	<10.0 U/mL	9.2	9.6	8.8
aCL IgM, U/mL	<10.0 U/mL	5.2	5.3	3.4
aβ2GPI IgG, U/mL	<10.0 U/mL	4.0	3.7	2.6
Aβ2GPI IgM, U/mL	<10.0 U/mL	3.2	3.9	2.6
LAC		Negative	Undosed (acute thrombotic event)	Undosed (VKA therapy)
P/F, mmHg/%	>300 mmHg/%	490	371	–
pCO_2_, mmHg	42 mmHg	41	34	–
pH	7.42 ± 0.2	7.45	7.41	–
HS-cTn	<0.014 μg/L	–	0.004	–

CRP, C-reactive protein; ALT, alanine aminotransferase; AST, Aspartate aminotransferase; RF, rheumatoid factor; anti-CCP, anti-citrulline antibodies; ANA, anti-nuclear antibodies; aCL, anti-cardiolipin; aβ2GPI, anti-β2 glicoprotein-I; LAC, Lupus Anticoagulant; HS-cTn, high-sensitivity cardiac troponin.

**Figure 1:**
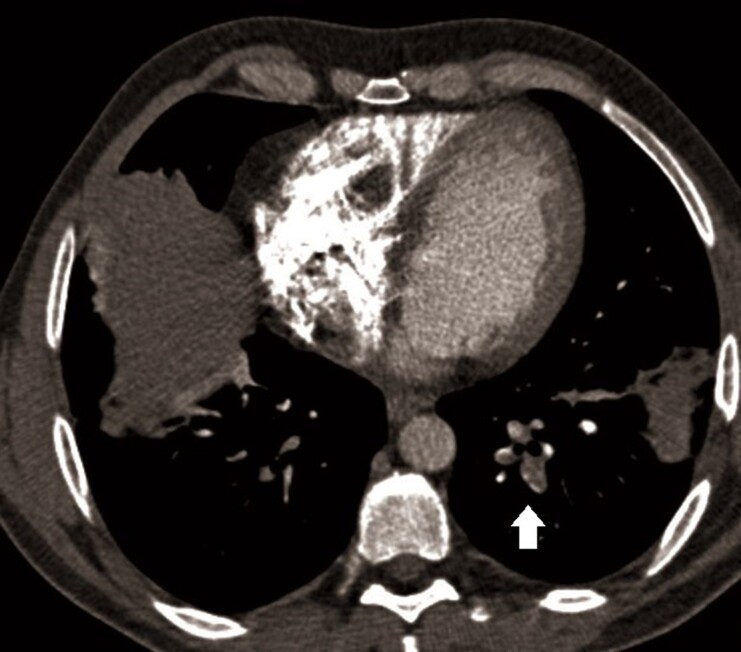
Thorax CT scan with iodinated contrast, axial view. The white arrow indicates PE in the left lower lobe posterior basal sub-segmental pulmonary artery.

**Figure 2:**
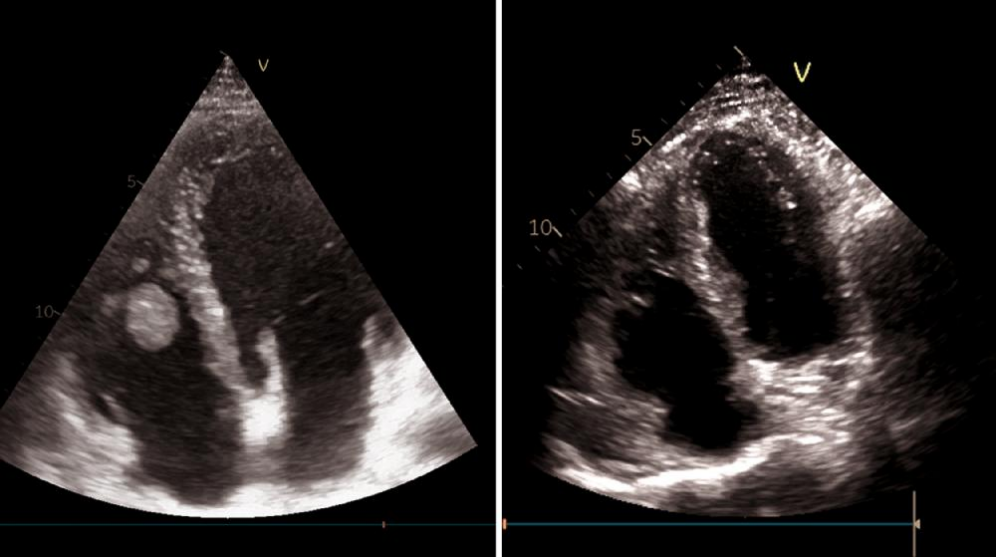
On the left: 2D TTE (apical four-chamber view) performed after PE diagnosis showing a large thrombotic mass in the right ventricle. On the right: 2D TTE (apical four-chamber view) performed after 5 months of target therapy. Thrombosis was not visible anymore.

**Figure 3:**
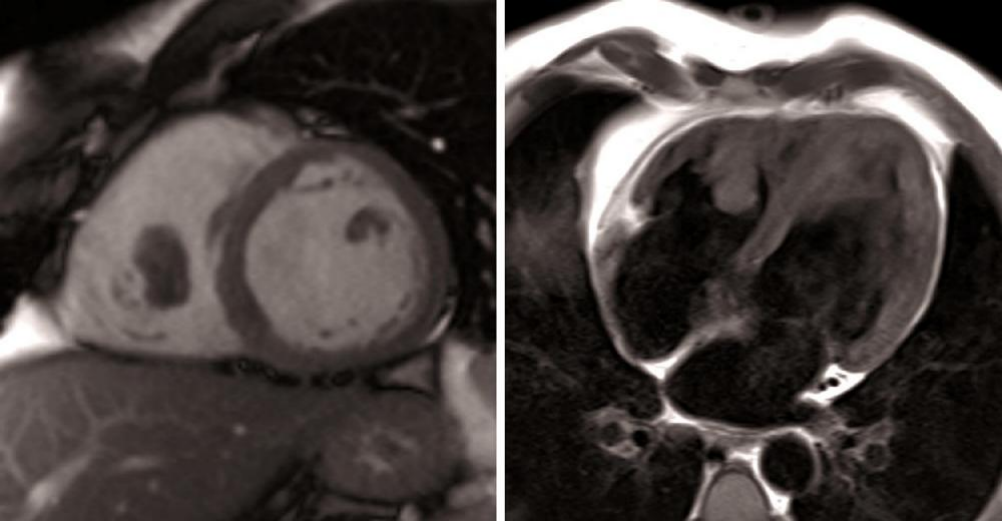
CMR showing thrombosis in the right ventricle. On the left: gradient echo sequences, short axis view. On the right side: T1-weighted sequence Double Inversion Recovery, four-chamber view.

To differentiate between different causes of haemoptysis, such as arteriovenous malformation, haemorrhagic alveolitis, cavitary tuberculosis, lung cancer, or pulmonary embolism (PE) (low Well’s score, but high D-dimer and possible endothelial dysfunction due to Behcet’s disease), we performed a chest CT scan with contrast enhancement. The CT scan revealed a small thrombus in the left lower lobe posterior sub-segmental pulmonary artery and a basal triangular left pulmonary consolidation consistent with pulmonary infarction (*Figure 1*). A lower extremity venous ultrasound showed no signs of deep vein thrombosis (DVT). As a result, a trans-thoracic echocardiogram (TTE) was performed. The exam revealed slightly reduced systolic function in both ventricles and a floating, hyperechoic, and well-shaped mass measuring 31 × 18 mm, likely of thrombotic origin, attached by a peduncle to the free wall of the right ventricle (see Supplementary material online, *Videos 1* and *2*). No signs of PE were found on the echocardiogram (*Figure 2*, *Table 2*). A cardiac magnetic resonance (CMR) characterized the exophytic mass in the right ventricle, considering this as an atypical site for thrombosis. The well-defined shape, the dynamic motion, and the absence of late gadolinium enhancement during contrast phases confirmed the diagnosis of thrombosis, ruling out other possible diagnoses such as myxoma, fibroma, or metastasis. Additionally, the right ventricle ejection fraction was found slightly reduced (46%) (*Figure 3*). An abdominal CT scan was performed to exclude other atypical thrombosis sites due to BD, malignancy, or other unpredictable complications, and it came back negative.

**Table 2:** **Echocardiographic measurements at PE diagnosis, focused on RV dimensions and systolic function of both ventricles** ^6^

Echocardiographic data	Normal values	Results
LVEF, %	>52%	50
RV dimension, mm	Basal < 42 mm	Basal 41
	Mid-cavity < 35 mm	Mid-cavity 35
TAPSE, mm	>17 mm	23 mm
RV FAC, %	>35%	33.3
PE signs	60/60 sign	Negative
	D shape	Negative
	High PAPs	40 mmHg

LVEF, left ventricular ejection fraction (Simpson method); RV, right ventricle; TAPSE, tricuspid annular plane systolic excursion; FAC, RV fractional area change; PE, pulmonary embolism.

During the patient’s hospitalization, we prescribed a thrombophilia screen, to rule out any clinical overlap between autoimmune or prothrombotic mutation and BD, despite it is known its endothelial involvement. DNA testing was conducted for factor V Leiden and prothrombin G20210A mutations, but the results were negative. Protein C and S activity were also tested before starting anticoagulant treatment, but these values may have been misleading due to the acute phase of the illness. Therefore, a new test for protein C and S activity will be recommended once the patient has fully recovered from the acute phase and has been symptom-free for at least six months.^7^ Antiphospholipid antibodies were not detected, and a test for lupus anticoagulant (LAC) was not performed due to the high rate of false positives and negatives during the acute phase of a thrombotic event.^7^

The case was discussed with the cardiac surgery unit, but the consultant surgeon advised against thrombectomy due to the high risk and likelihood of recurrence.^8^ Thrombolysis was not considered because the patient was haemodynamically stable, however, there is little experience with thrombolysis efficacy in BD. As a result, on the hypothesis of an inflammatory genesis of the thrombosis, we prescribed a treatment plan consisting of prednisone 50 mg per day, azathioprine 100 mg per day, and warfarin, with a target INR of 2.5–3, along with bridging therapy using low-molecular-weight heparin. We chose to use a vitamin K antagonist (VKA) instead of direct oral anticoagulants (DOACs), due to the lack of current strong recommendations for using DOACs in cases of intracardiac thrombosis, particularly in cases with an inflammatory cause.^9^

After the patient was discharged, a TTE was performed every 3 weeks, and we observed a gradual decrease in the mass size, without any side effects from the prescribed therapy. After 5 months, the mass had completely dissolved, and there were no recurring symptoms of BD (*Figure 2*). The rheumatologist confirmed that the patient’s immunosuppressive and anticoagulant therapy should remain unchanged for at least 6 months following the PE. If the patient remains asymptomatic, further adjustments to the treatment plan may be considered.

## Discussion

Vascular involvement in BD can affect from 7% to 46% of patients and the exact pathogenesis is still unclear.^2^ It is believed that environmental factors, such as infective agents or dysbiosis, and a genetic predisposition can lead to an immunological response resulting in hyperactivated neutrophils and perivascular infiltration. Recent studies have shown that neutrophil activation can promote fibrinogen oxidation and thrombosis formation in BD. This, in turn, can lead to an inflammatory cascade, as coagulative components such as fibrinogen, thrombin, factor Xa, and factor VIIa are amplified. An increased activity of Von Willebrand factor has also been observed.^8,10^ Cardiac involvement in BD is less common, with a prevalence ranging from 1% to 6%.^5^ Intracardiac thrombosis is rare, affecting mainly the right cardiac chambers, and especially the right ventricle. This type of thrombosis is usually caused by ‘in transit thrombi’ originating from peripheral DVT. However, it can also be caused by primary intracardiac processes, such as right heart wall impairment, devices, and atrial fibrillation.^11^ Thrombophilia, which can be caused by genetic mutations or paraneoplastic conditions, may also contribute to right heart thrombosis. However, there is currently no evidence to suggest that any specific thrombophilia factors are associated with the thrombotic tendency observed in BD.^12^ This suggests that the pathogenesis of thrombosis in BD is independent of any coagulation abnormalities. In the case of our patient, there was no evidence of abnormal myocardial motion or arrhythmias, and no auto-antibodies of autoimmune disease, genetic mutations, or neoplastic diseases were detected. Additionally, while corticosteroids can sometimes lead to prothrombotic conditions, our patient had only received a short course of prednisone at a low dose, making it unlikely to have contributed to the development of the thrombus. Therefore, it is likely that this atypical thrombosis was caused by BD, considering its pro-coagulant predisposition due to vasculitis.

Treatment of thrombotic complications in BD patients typically involves immunosuppression with corticosteroids, azathioprine, cyclosporin A, or cyclophosphamide, in addition to anticoagulation.^13^ However, the EULAR group does not provide guidance on the duration of anticoagulant therapy. Studies have shown that anticoagulation and immunosuppressant drugs together are more effective than anticoagulation alone.^12^ Vitamin K antagonists are commonly used, but DOACs may be a promising alternative in the future. However, there is limited data^14^ on their use in BD and inflammatory thrombosis, with little use in the treatment of DVT.^15^ There are currently no established criteria for discontinuing anticoagulant therapy, but it should be continued at least during the active phase of the disease and potentially lifelong in patients with refractory vascular involvement. The decision to continue anticoagulation should be based on the risk-benefit ratio.^13^

In conclusion, PE is a common cardiovascular disease that can sometimes be caused by rare conditions. Thrombosis may be the only clinical manifestation of primary or recurrent events in BD, and the site of origin may be atypical,^16^ as demonstrated in our case report. Thrombosis may indicate a high level of inflammation or an inadequate response to immunosuppressive therapy, which should be balanced with appropriate immunosuppressive treatment and anticoagulation. Some cases have shown that anticoagulation alone may not be effective in promoting regression or resolution of thrombosis, and the addition of immunosuppressive therapy may be necessary.^14^ This could be due to the presence of inflammation-promoting thrombotic events.

However, further studies are needed to better understand the underlying mechanisms and develop effective treatment strategies in this relatively unexplored field.

Our case report contributes to expanding knowledge and sharing experiences in the treatment and follow-up of patients with BD and cardiovascular involvement.

## Lead author biography


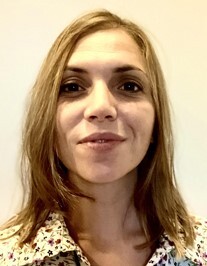


Valeria Ambrosino is 31 years old; she has just end the internal medicine post-graduating school of ‘La Sapienza’ University, resident in Policlinico Umberto I, Rome, Italy.

## Supplementary Material

ytae467_Supplementary_Data

## Data Availability

Data will be shared on reasonable request to the corresponding author with the permission of the patient.
